# Skeletal stem/progenitor cell-derived rather than osteoblast-derived IGF2 supports the development and homeostasis of skeletal system via STAT3

**DOI:** 10.7150/ijbs.112605

**Published:** 2025-04-28

**Authors:** Tairan Wang, Kunao Zhu, Yi Tang, Yuxing Xia, Qian Zhang, Rong Cong, Chaoqun Pan, Feiwu Kang

**Affiliations:** Shanghai Engineering Research Center of Tooth Restoration and Regeneration & Tongji Research Institute of Stomatology & Shanghai Tongji Stomatological Hospital and Dental School, Tongji University, Shanghai, China.

**Keywords:** Silver-Russell Syndrome, skeletal stem/progenitor cell, insulin-like growth factor 2, skeletal system, signal transducers and activators of transcription 3

## Abstract

**Rationale:** Skeletal deformities are characteristic manifestations of Silver-Russell Syndrome with mutations in insulin-like growth factor 2 (*IGF2*) gene serving as the most prevalent genetic abnormality. However, the mechanism by which *IGF2* influence bone development and homeostasis remains unclear. Besides, the expression pattern of *Igf2* and the local cells-derived* Igf2* at cellular level within bone microenvironment also lacked research.

**Methods:** ScRNA-seq analysis was obtained from Gene Expression Omnibus (GEO) and analyzed to discover the expression pattern of *Igf2.* Then, mice models of specific deletion of *Igf2* in three different cell types by crossing *Igf2 flox* with *Osx^cre^*, *Prx1^cre^* and *Ocn^cre^* mice respectively were established. The skeletal systems of these mice were analyzed, along with the *in vitro* osteogenic capacity of their bone marrow-derived mesenchymal stem cells (BMSCs). Lastly, RNA-sequencing (RNA-seq) was carried out to elucidate the underlying mechanisms, and administration of an agonist targeting the key molecule was also undertaken.

**Results:** *Igf2* was enriched in skeletal stem/progenitor cells (SSPCs) rather than osteoblasts and we have observed deformities in the femur and other skeletons in *Osx^Cre^; Igf2^fl/-^, Prx1^Cre^; Igf2^fl/-^* but not *Ocn^Cre^; Igf2^fl/-^* mice and the deformities were induced by a reduction in osteogenesis with minimal changes in osteoclastogenesis. It is further revealed that *Igf2* deletion impaired the osteogenesis via STAT3 and pharmacological activation of STAT3 could reverse the skeletal deformities.

**Conclusions:** In summary, these finding reveals that IGF2 derived from SSPCs, rather than osteoblasts, supports the development and homeostasis of skeletal system via STAT3 signaling and targeting STAT3 might be a promising therapeutic strategy for SRS-related skeletal deformities.

## Introduction

Silver-Russell Syndrome (SRS) is a rare disorder characterized by prenatal and postnatal growth retardation-related symptoms 1, with an incidence range of 1 in 30,000 to 100,000. The primary clinical manifestations encompass growth retardation, bodily asymmetry, aberrant fontanel development, and micrognathia, intricately linked to the development and remodeling of the skeletal system throughout the body 2-4. Numerous researchers have studied on the causes of the disease and found approximately 60% SRS cases could be attributed to genetic abnormalities 5. Interestingly, individuals with mutations in the insulin-like growth factor 2 (*IGF2*) exhibited more obvious developmental and skeletal abnormalities 1,6,7. Although these SRS-related bone deformities exert tremendous negative impacts on the patient's physical and mental health, the underlying mechanisms remain largely elusive. Hence, it is of great importance to clarify the effects of IGF2 on skeletal development and homeostasis to explore potential treatment methods.

IGF2, a member of the insulin-like growth factor (IGF) family, plays a crucial role in embryonic and postnatal growth and its deficient expression can lead to SRS 8-10. Besides, *Igf2* knockout mice exhibited a reduction in birth weight by about 60% and their long bone development is also significantly affected 11,12, indicating its indispensable role in overall development as a hormone in circulation. Meanwhile, IGF2 can also influence the development and homeostasis of local tissues via autocrine and paracrine: the *ex vivo* long bones of global knockout *Igf2* mice still grew slowly with structural abnormalities without the influence of IGF2 in blood circulation 12. Collectively, local cells-secreted IGF2 in skeletal system may well affect the growth and homeostasis of bones. Nevertheless, the identification of the crucial cell clusters responsible for the SRS-related skeletal deformities due to *Igf2* ablation has not been fulfilled yet.

The development and homeostasis of skeletal system involves multiple cell types and their intricate interactions 13,14. Particularly, the balance between bone-forming osteoblasts (OBs) and bone-resorbing osteoclasts (OCs) plays a crucial role in the process. Developmental bone deformities and decreased bone mass can be the result of impaired bone formation, excessive bone resorption or both. However, different interpretations of the role of IGF2 in osteogenesis has emerged. Controversial data call for further elaboration on the expression and function of *Igf2* at cellular level. Given that germline Cre lines are widely utilized for conditional gene knockout, it is possible to verify the main cell clusters which mainly secrete IGF2 within bone microenvironment and clarify their function.

In this study, firstly the single-cell RNA sequencing (scRNA-seq) data were reanalyzed to investigate the expression of *Igf2* among all BM-resident cells and explore the expression pattern along the trajectory of osteogenic lineage differentiation and complex interactions among mesenchymal cells. The results showed *Igf2* was more enriched in skeletal stem/progenitor cells (SSPCs) compared with mature osteoblasts*.* Then, we established 3 mice model to delete *Igf2* in different lineage cells, namely *Prx1^Cre^; Igf2^fl/-^, Osx^Cre^; Igf2^fl/-^* and *Ocn^Cre^; Igf2^fl/-^*, to identify the function of *Igf2* at cellular level and found that the quality and quantity of craniofacial and long bones were reduced in *Prx1*-dependent and* Osx*-dependent, but not *Ocn*-dependent *Igf2* deletion mice. Lastly, RNA-seq analysis demonstrated that SSPC-derived IGF2 influenced the development and homeostasis of skeletal system via STAT3. Furthermore, we discovered the administration of the agonist of STAT3, colivelin, reversed the effects of *Igf2* depletion on osteogenesis both *in vivo* and *in vitro*.

## Results

### *Igf2* is mainly expressed in mesenchymal cells in bone microenvironment

**Figure 1:**
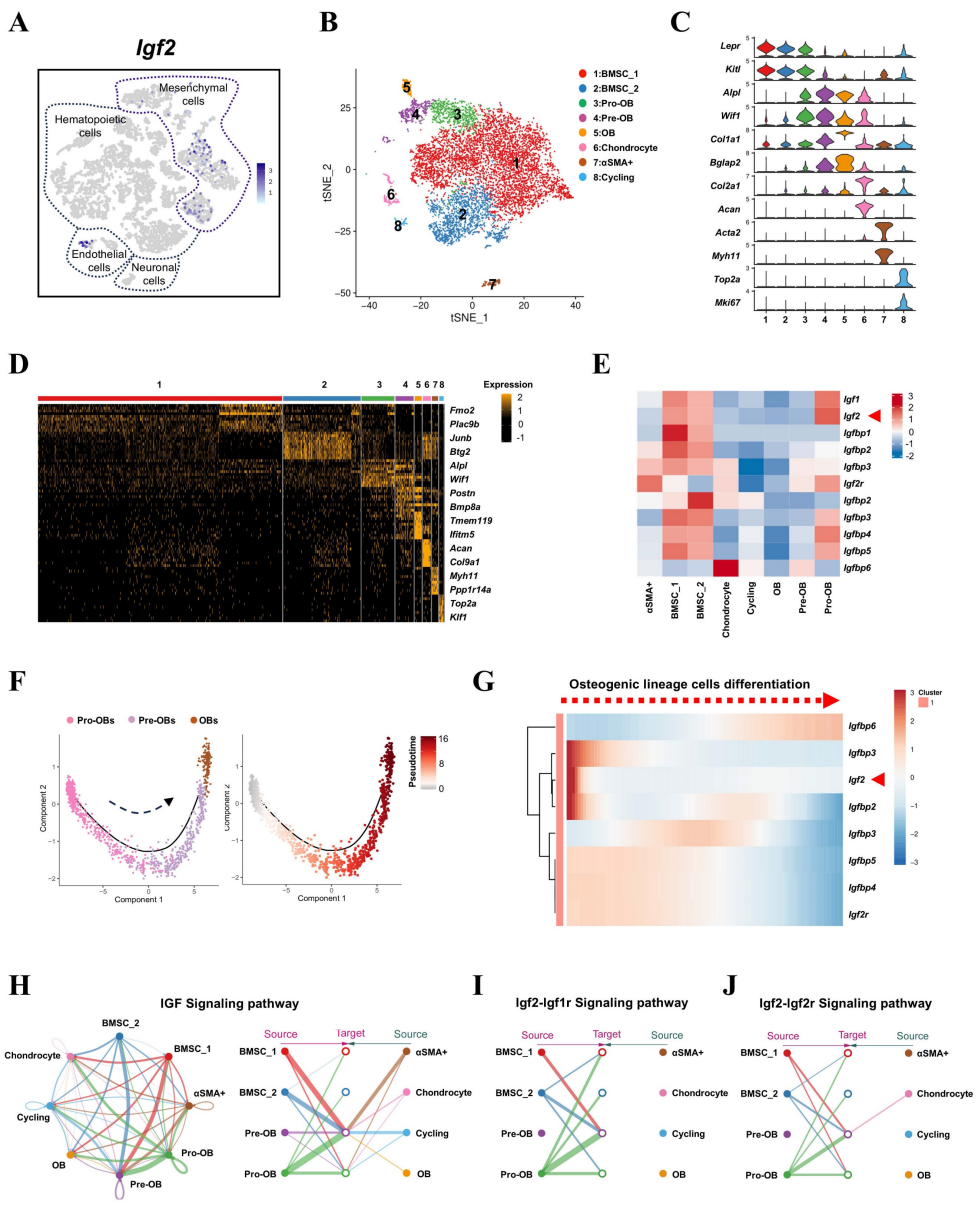
*Igf2* is expressed in mesenchymal cells among all BM-resident cell types. **A** *Igf2* expression in different BM-resident cell types by scRNA-seq. **B** t-SNE plot showing integrated analysis of mesenchymal cells (defined as PRX1^+^ or LEPR^+^ cells in raw data) and cells stained with different colors by clusters. **C** Violin plot showing the expression of feature genes for each cluster. **D** Heatmap showing the relative expression levels of 10 most significant markers for each cluster and 2 representative genes of each cluster shown on the right end. **E** Heatmap showing the expression of *Igf2, Igf1* and other binding proteins of IGF among different clusters of mesenchymal cells. **F** Single-cell trajectory of osteogenic lineage cells in a pseudo-temporal order from left to right. **G** Heatmap showing the dynamic changes of genes expression in a pseudo-temporal order from left to right. **H** Cell chat analysis showing the intercellular communication network of IGF signaling pathway among mesenchymal cells. **I** Cell chat analysis showing the intercellular communication network of IGF2-IGF1R and IGF2-IGF2R signaling pathway among mesenchymal cells.

The misexpression of *Igf2* accounted for one-third of BWS cases and two-thirds of SRS cases, but what role does the local expression of *Igf2* in bone microenvironment plays remains elusive 15. To investigate the distinct expression pattern of *Igf2* in bone microenvironment, we first reanalyzed the single-cell transcriptomes of all BM-resident cells published by a previous study 16. Compared with other cell types, *Igf2* was mainly expressed in mesenchymal cells (Fig. 1A). To further distinguish its expression in various types of mesenchymal cells, especially those closely related with bone formation and homeostasis, another single-cell RNA sequencing (scRNA-seq) dataset of the skeletal lineage cells was selected 17. As a result, eight clusters of cells were defined (Fig. 1B) according to their characteristic expression. Specifically, we identified subsets including BMSCs (cluster 1 and 2), Pro-OBs (cluster 3), Pre-OBs (cluster 4), OBs (cluster 5), Chondrocytes (cluster 6), αSMA cells (cluster 7) and Cycling cells (cluster 8) (Fig. 1C, D). Next, we analyzed the expression of *Igf2*, *Igfbps* and *Igf2r* among different clusters and the results showed *Igf2* was enriched in earlier stage cells (SSPCs), like BMSCs and Pro-OBs and other genes showed multiple expression patterns (Fig. 1E& Fig S1). Then, a single-cell trajectory of OB lineage cells was formed based on a pseudo-temporal order, which indicated Pro-OBs differentiated into OBs (Fig. 1F). In addition, the expression pattern of *Igf2* showed high expression at earlier stage according to the trajectory (Fig. 1G). To further investigate the mechanism by which IGF signaling pathway functions within mesenchymal cells, cell chat analysis was performed utilizing the scRNA-seq data. Interestingly, Pre-OBs exhibited little interaction among themselves and cells at late differentiation stage (cluster 5, 6, 7, 8) in Igf2-Igf1r and Igf2-Igf2r signaling pathway, while considerable interactions existed among these cells in IGF signaling pathway. Collectively, these findings indicate that *Igf2* is primarily expression in mesenchymal cells in bone microenvironment and the SSPC-secreted IGF2 may exert significant effects on Pre-OBs and bone homeostasis.

### Deleting *Igf2* in pro-osteoblasts leads to skeletal deformities due to decreased bone formation

To investigate the role of *Igf2* in pro-osteoblasts on the osteogenesis, we deleted *Igf2* in pro-osteoblasts by crossing male *Igf2 flox* mice with female *Osx^Cre^* mice (*Osx^Cre^; Igf2^fl/-^*). The qPCR analysis revealed a significant reduction of approximately 80% in *Igf2* levels in BMSCs from *Osx^Cre^; Igf2^fl/-^* mice (CKO referring to *Osx^Cre^; Igf2^fl/-^* in this section), when compared to littermate controls (Ctrl referring to *Osx^Cre^* in this section) (Fig. S2A). Plus, the osteogenesis-related genes, including *Col1a1*, *Alp*, *Runx2*, *Sp7* and *Ocn*, were also tested and all downregulated in CKO group.

**Figure 2:**
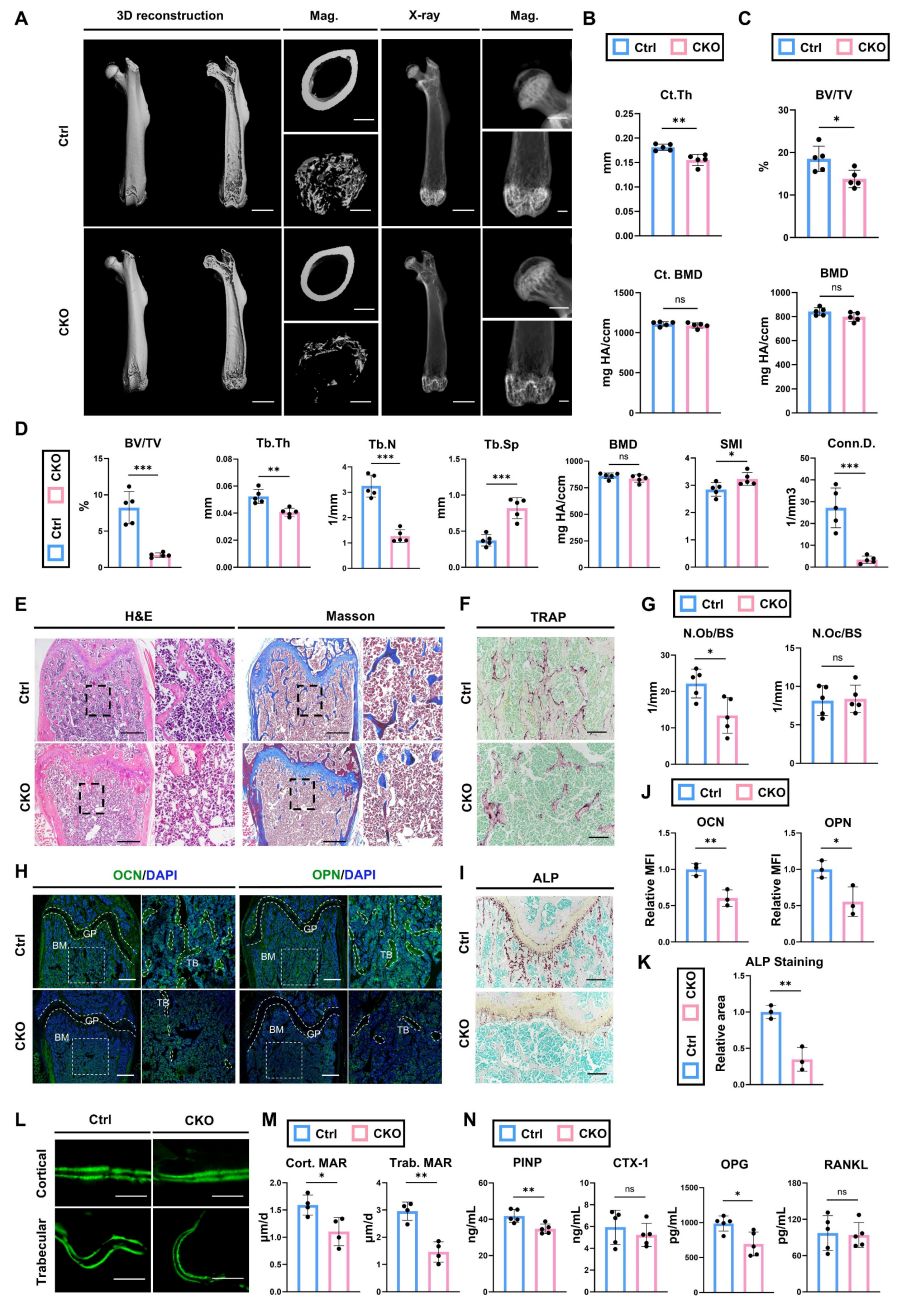
Deletion of Igf2 in pro-osteoblasts induces skeletal deformities by reducing bone formation. **A** Representative micro-CT images of femur and its magnifying views of the femur sections from 12-wk-old male *Osx^Cre^; Igf2^fl/-^* mice and littermate controls. Scale bar=2mm for 3D reconstruction and X-ray; 500μm for magnified views. **B** Statical analysis of micro-CT of cortical thickness (Ct. Th) and cortical bone mineral density (Ct. BMD) (n=5 per genotype). **C** Statical analysis of micro-CT of bone volume ratio (BV/TV) and bone mineral density (BMD) of femoral head (n=5 per genotype). **D** Statical analysis of micro-CT of trabecular bone volume ratio (BV/TV), trabecular thickness (Tb. Th), trabecular number (Tb. N), trabecular spacing (Tb. Sp), bone mineral density (BMD), structure model index (SMI) and connectivity density (Conn.D) (n=5 per genotype). **E** Representative H&E and Masson trichrome staining images of the femur sections from 12-wk-old male *Osx^Cre^; Igf2^fl/-^* mice and littermate controls. Scale bar=500μm. **F** Representative Tartrate-resistant acid phosphatase (TRAP) staining images of the femur sections from 12-wk-old male *Osx^Cre^; Igf2^fl/-^* mice and littermate controls. Scale bar=200μm. **G** Quantification of osteoblast number /bone surface (N.Ob/BS) and osteoclast number /bone surface (N.Oc/BS) of the femur sections from 12-wk-old male *Osx^Cre^; Igf2^fl/-^* mice and littermate controls according to E&F (n=5 per genotype). **H** Representative Immunofluorescence staining and its magnifying views of osteocalcin (OCN) and osteopontin (OPN) of the femur sections from 12-wk-old male *Osx^Cre^; Igf2^fl/-^* mice and littermate controls. Scale bar=500μm. **I** Representative Alkaline Phosphatase (ALP) staining images of the femur sections from 12-wk-old male *Osx^Cre^; Igf2^fl/-^* mice and littermate controls. Scale bar=200μm. **J** Statical analysis of the mean fluorescent intensity (MFI) of H (Ctrl set as 1, n=3 per genotype). **K** Statical analysis of ALP staining area of I (Ctrl set as 1, n=3 per genotype). **L, M** Calcein double labeling in trabecular and cortical bones and representative images (L) and statical analysis of each group (M, n=4 per genotype) were shown. Scale bar=50μm. **N** Serum ELISA of pro-peptide of type I procollagen (PINP), C-terminal telopeptides of type I collagen (CTX-I), receptor activator NF-kappa B ligand (RANKL) and osteoprotegerin (OPG) (n=5 per genotype). Data are presented as mean ± SEM Statistical significance is denoted as follows: *p < 0.05, **p < 0.01, ***p < 0.001.

Next, micro-CT was performed to analyze the bone parameters of femurs of 12-wk-old *Osx^Cre^; Igf2^fl/-^* and littermate controls (Fig. 2A). The analyses showed that cortical thickness (Ct. Th) in CKO group was significantly lower than that in Ctrl group, while the cortical bone mineral density (BMD) was not significantly different (Fig. 2B). Micro-CT analyses on femoral head also showed similar trends: significant decreased bone volume ratio (BV/TV) in CKO compared with Ctrl group (Fig. 2C). For distal femur metaphysis, CKO group exhibited a significant reduction in BV/TV, trabecular thickness (Tb.Th), trabecular number (Tb.N) and connectivity density (Conn.D.) as well as a significant increase in trabecular spacing (Tb.Sp) and structure model index (SMI) compared to Ctrl mice (Fig. 2D). These data indicated that CKO group showed inferior quality and quantity of femurs compared with Ctrl group. Next, we continued to evaluate the bone formation and bone resorption ability between two groups to figure out the reason of decreased bone volume in CKO group. Osteoblast number /bone surface (N.Ob/BS) analyzed by H&E staining showed a significant decrease in CKO group (Fig. 2E, G), while there was no significant difference in osteoclast number /bone surface (N.Oc/BS) analyzed by TRAP staining between two groups (Fig. 2F, G). Osteogenesis-related proteins OCN and OPN were also significantly decreased in CKO group (Fig. 2H, J). Likewise, ALP staining also showed a similar trend (Fig. 2I, K). Then, calcein double labeling was conducted to evaluate dynamic bone formation and significant slower MAR was witnessed in both cortical and trabecular bones from CKO group (Fig. 2L, M). Lastly, the serum level of PINP, CTX-1, OPG and RANKL were also detected. The results demonstrated that bone formation marker P1NP and OPG were significantly decreased in CKO group whereas bone resorption marker CTX-1and RANKL showed no evident difference (Fig. 2N). Plus, bone defects and skeletal deformities were also observed in other parts, like skull and condyle of 12-wk-old CKO group (Fig. S3). Besides, the CKO group exhibited decreased bone formation from much earlier developmental stages: analyses of the femurs of 2-wk-old and 4-wk-old CKO group all showed significantly impaired osteogenic ability, with little change in osteoclasts function (Fig S4). Collectively, these data indicated that deletion of *Igf2* in pro-osteoblasts causes skeletal deformities via reducing bone formation, while left bone resorption ability almost intact.

### Deletion of *Igf2* in BMSCs induces skeletal deformities by reducing bone formation

**Figure 3:**
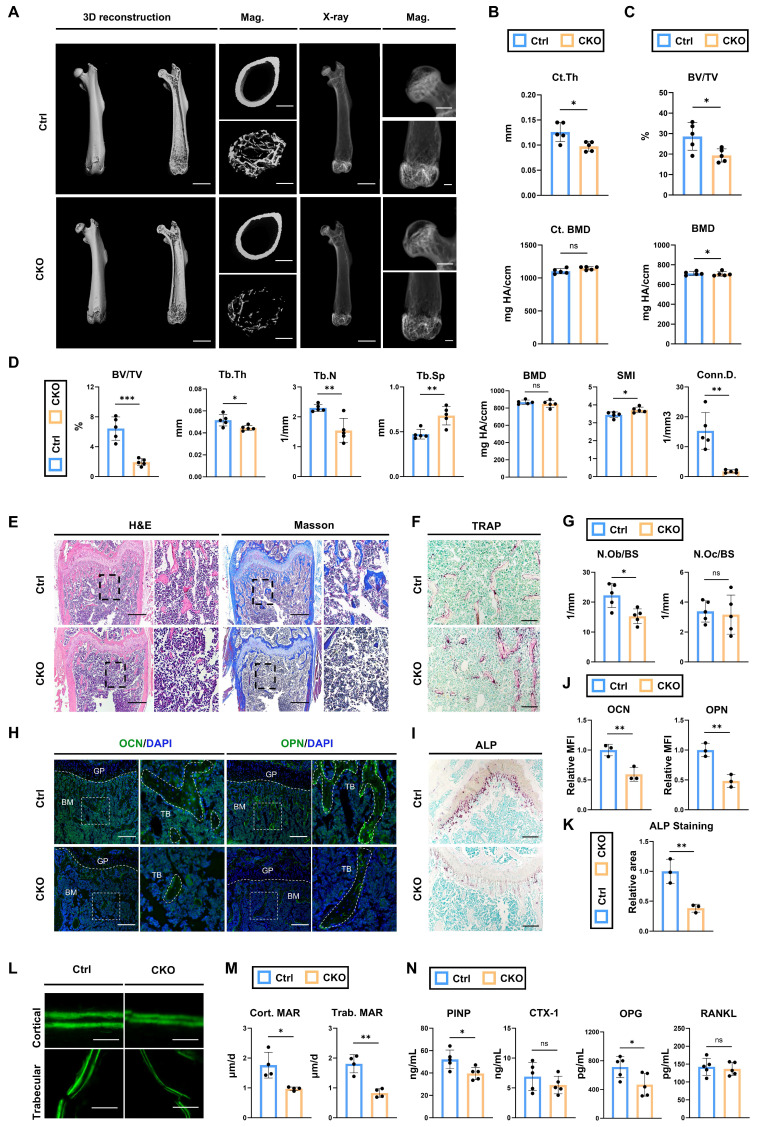
Deletion of *Igf2* in BMSCs results in skeletal deformities by reducing bone formation. **A** Representative micro-CT images of femur and its magnifying views of the femur sections from 12-wk-old male *Prx1^Cre^; Igf2^fl/-^* mice and littermate controls. Scale bar=2mm for 3D reconstruction and X-ray; 500μm for magnified views. **B** Statical analysis of micro-CT of cortical thickness (Ct. Th) and cortical bone mineral density (Ct. BMD) (n=5 per genotype). **C** Statical analysis of micro-CT of bone volume ratio (BV/TV) and bone mineral density (BMD) of femoral head (n=5 per genotype). **D** Statical analysis of micro-CT of trabecular bone volume ratio (BV/TV), trabecular thickness (Tb. Th), trabecular number (Tb. N), trabecular spacing (Tb. Sp), bone mineral density (BMD), structure model index (SMI) and connectivity density (Conn.D) (n=5 per genotype). **E** Representative H&E and Masson trichrome staining images of the femur sections from 12-wk-old male *Prx1^Cre^; Igf2^fl/-^* mice and littermate controls. Scale bar=500μm. **F** Representative Tartrate-resistant acid phosphatase (TRAP) staining images of the femur sections from 12-wk-old male *Prx1^Cre^; Igf2^fl/-^* mice and littermate controls. Scale bar=200μm. **G** Quantification of osteoblast number /bone surface (N.Ob/BS) and osteoclast number /bone surface (N.Oc/BS) of the femur sections from 12-wk-old male *Prx1^Cre^; Igf2^fl/-^* mice and littermate controls according to E&F (n=5 per genotype). **H** Representative Immunofluorescence staining and its magnifying views of osteocalcin (OCN) and osteopontin (OPN) of the femur sections from 12-wk-old male *Prx1^Cre^; Igf2^fl/-^* mice and littermate controls. Scale bar=200μm. **I** Representative Alkaline Phosphatase (ALP) staining images of the femur sections from 12-wk-old male *Prx1^Cre^; Igf2^fl/-^* mice and littermate controls. Scale bar=200μm. **J** Statical analysis of the mean fluorescent intensity (MFI) of H (Ctrl set as 1, n=3 per genotype). **K** Statical analysis of ALP staining area of I (Ctrl set as 1, n=3 per genotype). **L, M** Calcein double labeling in trabecular and cortical bones and representative images (L) and statical analysis of each group (M, n=4 per genotype) were shown. Scale bar=50μm. **N** Serum ELISA of pro-peptide of type I procollagen (PINP), C-terminal telopeptides of type I collagen (CTX-I), receptor activator NF-kappa B ligand (RANKL) and osteoprotegerin (OPG) (n=5 per genotype). Data are presented as mean ± SEM Statistical significance is denoted as follows: ns, not significant, *p < 0.05, **p < 0.01, ***p < 0.001.

As aforementioned in scRNA-seq analysis, *Igf2* in the cell clusters representing BMSCs also exhibited considerable expression levels. To investigate the bone phenotypes induced by *Igf2* deficiency in BMSCs, we crossed male *Igf2 flox* mice with female *Prx1^Cre^* mice (*Prx1^Cre^; Igf2^fl/-^*). Next, micro-CT was performed to analyze the bone parameters of femurs of 12-wk-old *Prx1^Cre^; Igf2^fl/-^* and littermate controls (Fig. 3A). Interestingly, micro-CT analyses showed similar trends between *Prx1^Cre^; Igf2^fl/-^*(CKO referring to *Prx1^Cre^; Igf2^fl/-^* in this section) and littermates (Ctrl referring to *Prx1^Cre^* in this section) as we observed in *Osx^Cre^; Igf2^fl/-^*. The analyses showed that Ct. Th in CKO group was significantly lower than that in Ctrl group, while BMD was not significantly different (Fig. 3B). In terms of femoral head, CKO group exhibited significant decreased BV/TV compared with Ctrl group (Fig. 3C). For distal femur metaphysis, a significant reduction in BV/TV, Tb.Th, Tb.N and Conn.D. and a significant increase in Tb.Sp and SMI were observed in CKO group compared with Ctrl group (Fig. 3D). These data indicated that CKO group showed inferior quality and quantity of femurs compared with Ctrl group. Next, we continued to evaluate the bone formation and bone resorption ability between two groups to figure out the reason of decreased bone volume in CKO group. N.Ob/BS showed a significant decrease in CKO group (Fig. 3E, G), while there was no significant difference in N.Oc/BS between two groups (Fig. 3F, G). Osteogenesis-related proteins OCN and OPN were also significantly decreased in CKO group (Fig. 3H, J). Likewise, ALP staining also showed a similar trend (Fig. 3I, K). Then, calcein double labeling was conducted to evaluate dynamic bone formation and significant slower MAR was witnessed in both cortical and trabecular bones from CKO group (Fig. 3L, M).

Lastly, the serum level of PINP, CTX-1, OPG and RANKL were also detected. The results demonstrated that bone formation marker P1NP and OPG were significantly decreased in CKO group whereas bone resorption marker CTX-1and RANKL showed no evident difference (Fig. 3N). To sum up, these results demonstrated that deletion of *Igf2* in BMSCs also induces skeletal deformities like what we observed in* Osx^Cre^; Igf2^fl/-^*, due to the impaired bone formation but not the enhanced bone resorption.

### Ablation of *Igf2* in osteoblasts does not lead to severe malformations with similar bone formation and resorption abilities

**Figure 4:**
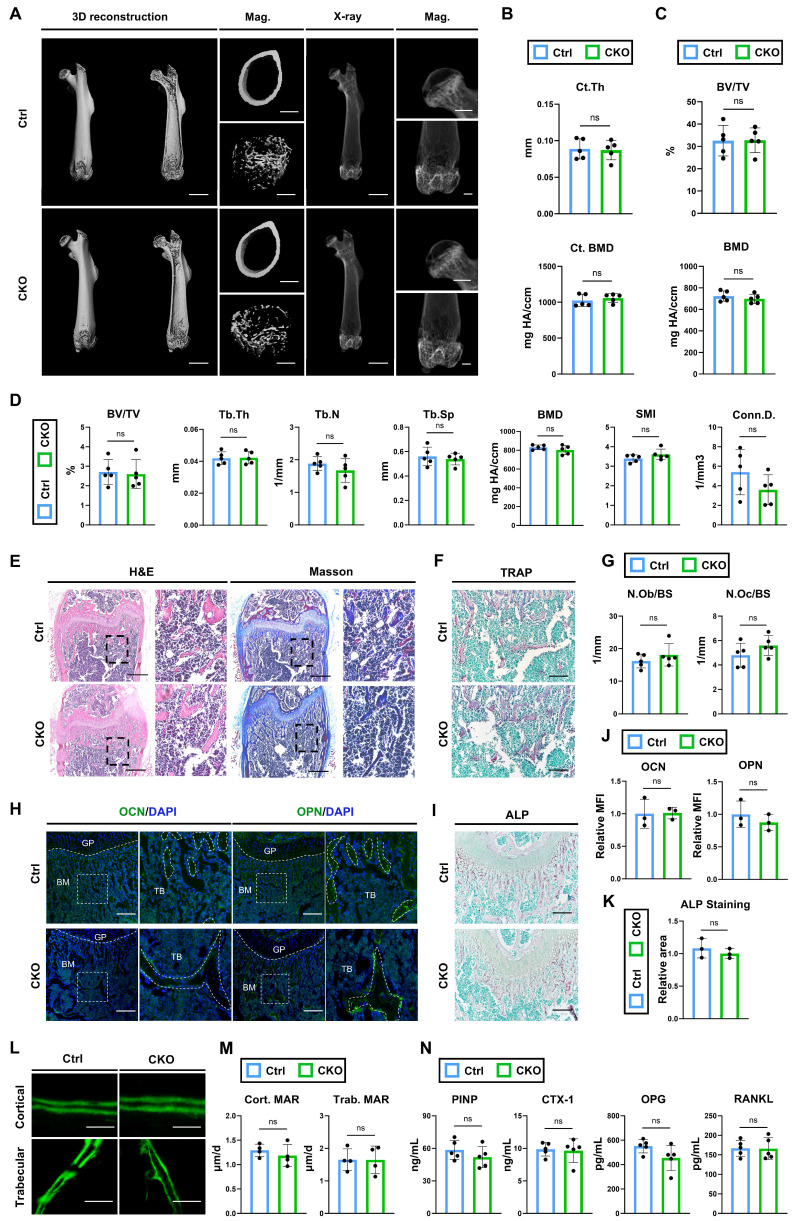
Ablation of *Igf2* in osteoblasts does not cause significant bone deformities. **A** Representative micro-CT images of femur and its magnifying views of the femur sections from 12-wk-old male *Ocn^Cre^; Igf2^fl/-^* mice and littermate controls. Scale bar=2mm for 3D reconstruction and X-ray; 500μm for magnified views. **B** Statical analysis of micro-CT of cortical thickness (Ct. Th) and cortical bone mineral density (Ct. BMD) (n=5 per genotype). **C** Statical analysis of micro-CT of bone volume ratio (BV/TV) and bone mineral density of femoral head (BMD) (n=5 per genotype). **D** Statical analysis of micro-CT of trabecular bone volume ratio (BV/TV), trabecular thickness (Tb. Th), trabecular number (Tb. N), trabecular spacing (Tb. Sp), bone mineral density (BMD), structure model index (SMI) and connectivity density (Conn.D) (n=5 per genotype). **E** Representative H&E and Masson trichrome staining images of the femur sections from 12-wk-old male *Ocn^Cre^; Igf2^fl/-^* mice and littermate controls. Scale bar=500μm. **F** Representative Tartrate-resistant acid phosphatase (TRAP) staining images of the femur sections from 12-wk-old male *Ocn^Cre^; Igf2^fl/-^* mice and littermate controls. Scale bar=200μm. **G** Quantification of osteoblast number /bone surface (N.Ob/BS) and osteoclast number /bone surface (N.Oc/BS) of the femur sections from 12-wk-old male *Ocn^Cre^; Igf2^fl/-^* mice and littermate controls according to E&F (n=5 per genotype). **H** Representative Immunofluorescence staining and its magnifying views of osteocalcin (OCN) and osteopontin (OPN) of the femur sections from 12-wk-old male *Ocn^Cre^; Igf2^fl/-^* mice and littermate controls. Scale bar=200μm. **I** Representative Alkaline Phosphatase (ALP) staining images of the femur sections from 12-wk-old male *Ocn^Cre^; Igf2^fl/-^* mice and littermate controls. Scale bar=200μm. **J** Statical analysis of the mean fluorescent intensity (MFI) of H (Ctrl set as 1, n=3 per genotype). **K** Statical analysis of ALP staining area of I (Ctrl set as 1, n=3 per genotype). **L, M** Calcein double labeling in trabecular and cortical bones and representative images (L) and statical analysis of each group (M, n=4 per genotype) were shown. Scale bar=50μm. **N** Serum ELISA of pro-peptide of type I procollagen (PINP), C-terminal telopeptides of type I collagen (CTX-I), receptor activator NF-kappa B ligand (RANKL) and osteoprotegerin (OPG) (n=5 per genotype). Data are presented as mean ± SEM Statistical significance is denoted as follows: ns, not significant.

According to the scRNA analysis, osteoblast is neither the primary cell expressing *Igf2* nor does it have cellular interactions with SSPCs in terms of Igf2 signaling pathways (Fig. 1I, J). However, this analysis is confined to a specific time point and the skeletal conditions of osteoblast-specific conditional knockout mice provide more compelling evidence for verifying its function. Thus, we deleted *Igf2* in osteoblasts by crossing male *Igf2 flox* mice with female *Ocn^Cre^* mice (*Ocn^Cre^; Igf2^fl/-^*). Next, micro-CT was performed to analyze the bone parameters of femurs of 12-wk-old *Ocn^Cre^; Igf2^fl/-^* and littermate controls (Fig. 4A). In contrast to the results we observed in *Osx^Cre^; Igf2^fl/-^* and *Prx1^Cre^; Igf2^fl/-^*, micro-CT analyses showed similar femur conditions between *Ocn^Cre^; Igf2^fl/-^*(CKO referring to *Ocn^Cre^; Igf2^fl/-^* in this section) and littermates (Ctrl referring to *Ocn^Cre^* in this section). The Ct. Th and cortical BMD as well as the BV/TV and BMD of femoral heads of two groups showed no significant difference (Fig. 4B, C). For distal femur metaphysis, CKO group exhibited similar phenotypes in BV/TV, Tb.Th, Tb.N and Conn.D. as well as in Tb.Sp and SMI compared to Ctrl mice (Fig. 4D). These data indicated that CKO group showed similar bone parameters as Ctrl group. Next, the bone formation and bone resorption ability between two groups were evaluated. Both N.Ob/BS and N.Oc/BS showed no significant difference in between two groups (Fig. 4E~G). The expression of osteogenesis-related proteins OCN and OPN were similar between the two groups (Fig. 4H, J). Likewise, ALP staining also showed no significant difference (Fig. 4I, K). Then, calcein double labeling was conducted to evaluate the dynamic bone formation and both cortical and trabecular bones showed similar MAR between two groups (Fig. 4L, M). Lastly, the serum level of PINP, CTX-1, OPG and RANKL were also detected. The results demonstrated that bone formation marker P1NP and OPG and bone resorption marker CTX-1and RANKL showed no evident difference (Fig. 4N). Collectively, these data indicated that deletion of *Igf2* in osteoblasts does not lead to severe malformations and furthermore, there is no significant difference in the capacity of bone resorption and bone formation.

### Osx^+^ cells -derived *Igf2* enhances the osteogenesis of the CD200^+^ mesenchymal cells via JAK-STAT signaling pathway

**Figure 5:**
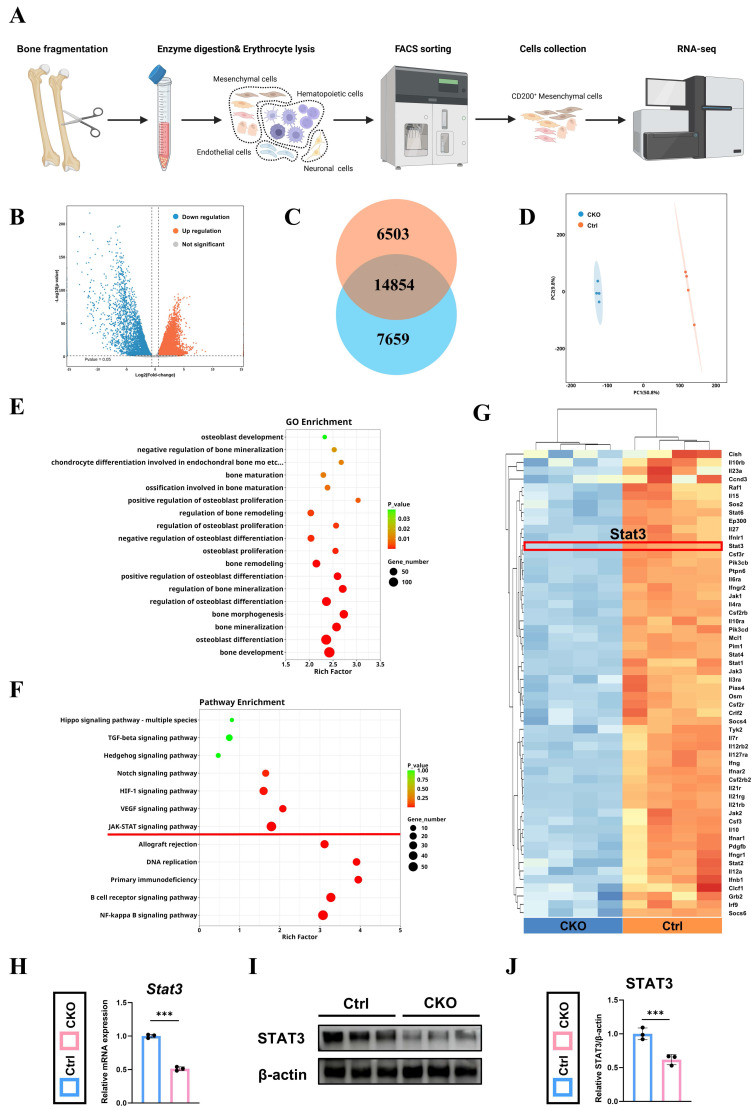
Osx^+^ cells-derived *Igf2* promotes the osteogenic ability of mesenchymal cells via JAK-STAT signaling pathway. **A** The schematic diagram showing the work flow of flow cytometry sorting and RNA sequencing. **B** The volcano plots showing the differentially expressed genes (DEGs) between the CD200 positive mesenchymal cells from *Osx^Cre^; Igf2^fl/-^* mice and littermate controls. **C** Venn diagrams illustrating the number of differentially expressed genes. **D** Principal component analysis of the two different groups (n=4 per genotype). **E, F** Gene Ontology-Biological Process (GO-BP) and Kyoto Encyclopedia of Genes and Genomes (KEGG) gene set enrichment analysis revealing serval pathways associated with bone generation. **G** The heat map showing the enriched genes in JAK-STAT signaling pathway. **H** RT-qPCR showing the relative expression of *Stat3* of different groups (n = 3 per group). **I, J** Western blotting indicating the expression levels of STAT3 in different groups (n = 3 per group). Data are presented as mean ± SEM Statistical significance is denoted as follows: ***p < 0.001.

To discover the underlying mechanism of the reduced osteogenic ability in *Osx^Cre^; Igf2^fl/-^* mice, we firstly separated the CD31^-^CD45^-^TER119^-^CD200^+^ cells (mesenchymal cells) from the femur fragments of both groups via flow cytometry sorting (details shown in Fig. S5), and then performed RNA-seq as illustrated in Fig. 5A. Differentially expressed genes (DEGs) were exhibited by volcano map (Fig. 5B), including 6503 upregulating and 7659 downregulating genes (Fig. 5C). In addition, the principal component analysis showed that two groups of samples were clustered separately, demonstrating that these two groups exhibited markedly different expression profile characteristics (Fig. 5D). GO-BP gene set enrichment analysis showed that plenty of osteoblast-related and bone-related processes were involved, indicating a dynamic change in osteogenesis (Fig. 5E). Meanwhile, KEGG analysis showed that the gene expression patterns were enriched in several osteogenic-related pathways, including JAK-STAT signaling pathway as a significant one (Fig. 5F). Then, a heat map was utilized to visualize all the JAK-STAT signaling pathway-associated genes that exhibited significant expression alterations and *Stat3*, a well-documented osteogenesis-related gene ranked top among genes within this pathway (Fig. 5G).

Lastly, RT-qPCR and western blotting were performed to further examine the results of RNA-seq and demonstrated that STAT3 was downregulated in CKO group at both transcriptional and protein levels (Fig. 5H~J). In summary, JAK-STAT signaling pathway and *Stat3* were remarkably downregulated in the mesenchymal cells form CKO group, which possibly related to its decreased osteogenesis.

### Pharmacological activation of STAT3 by colivelin partially rescues the decreased bone formation in *Osx^Cre^; Igf2^fl/-^* mice both *in vivo* and *in vitro*

**Figure 6:**
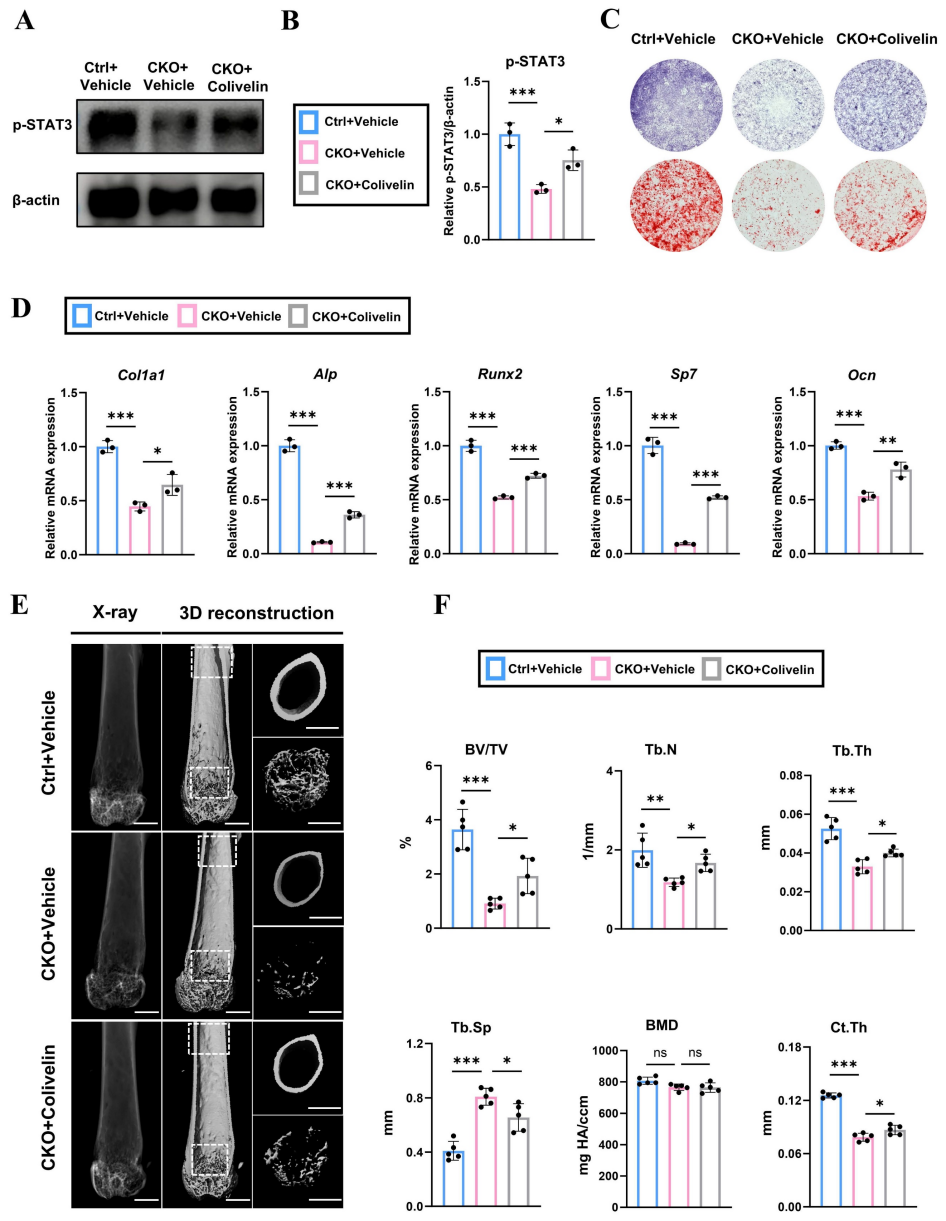
Administration of colivelin enhances the osteogenic ability of bone mesenchymal cells and partially prevents the bone loss in *Osx^Cre^; Igf2^fl/-^* mice. **A, B** Western blotting and its statical analysis illustrating the expression of p-STAT3 of BMSCs from *Osx^Cre^* (Ctrl) and *Osx^Cre^; Igf2^fl/-^* (CKO) mice with vehicle or STAT3 agonist colivelin (n=3 per group). **C** Representative images of alkaline phosphatase (ALP) staining and alizarin red staining (ARS) of BMSCs from *Osx^Cre^* (Ctrl) and *Osx^Cre^; Igf2^fl/-^* (CKO) mice with vehicle or STAT3 agonist colivelin. **D** RT-qPCR showing the relative expression of *Col1a1*, *Alp*, *Runx2*, *Sp7* and *Ocn* of BMSCs from *Osx^Cre^* (Ctrl) and *Osx^Cre^; Igf2^fl/-^* (CKO) mice with vehicle or STAT3 agonist colivelin (n=3 per group). **E** Representative micro-CT images of femur and its magnifying views of the femur sections from 8-wk-old male *Osx^Cre^; Igf2^fl/-^* mice and littermate controls after administration with vehicle or colivelin. Scale bar =1mm. **F** Statical analysis of micro-CT of trabecular bone volume ratio (BV/TV), trabecular thickness (Tb. Th), trabecular number (Tb. N), trabecular spacing (Tb. Sp), bone mineral density (BMD) and cortical thickness (Ct. Th) of samples in E (n=5 per genotype). Data are presented as mean ± SEM Statistical significance is denoted as follows: ns, not significant, *p < 0.05, **p < 0.01, ***p < 0.001.

To further verify the role of STAT3 in osteogenesis and test whether STAT3 could be applied as a therapy to rescue the SRS-related bone deformities via regulating bone remodeling, the agonist of STAT3, colivelin, was utilized to enhance the phosphorylation and activity of STAT3 both *in vivo* and *in vitro*. Firstly, we isolated BMSCs from 4-wk-old *Osx^Cre^; Igf2^fl/-^* and littermate mice and were then treated with or without colivelin at a concentration of 3μg/mL for 24h. Next, western blotting showed that the application of colivelin effectively enhanced the phosphorylation level of STAT3 decreased by *Igf2* deletion (Fig. 6A, B). ALP staining and alizarin red staining (ARS) were performed to measure the osteogenic capability of these BMSCs under the osteogenic induction medium coupled with colivelin at a concentration of 3ng/mL or vehicle (PBS) for 7 and 21 days respectively according to a previous article 18. The results demonstrated that colivelin also significantly enhanced the ALP positive area as well as the calcium nodules of the CKO group (Fig. 6C). Further, BMSCs from different groups were cultured in the osteogenic induction medium with colivelin or vehicle (PBS) for 3 days and RT-qPCR showed that colivelin also enhanced the expression of osteogenic genes *Col1a1*, *Alp*, *Runx2*, *Sp7* and *Ocn,* indicating the pharmacological activation of STAT3 by colivelin could reversed the decreased osteogenic ability of *Igf2* deletion *in vitro* (Fig. 6D).

Furthermore, to testify the function of colivelin *in vivo,* we intraperitoneally injected 4-wk-old *Osx^Cre^; Igf2^fl/-^* and littermate mice with colivelin at a concentration of 1 mg/kg or equal volume of vehicle for every other day for 4 weeks. Then, micro-CT was performed to evaluate the quality and quantity of the femurs of different groups (Fig. 6E). Statistical analysis demonstrated that colivelin administration rescued the decreased BV/TV, Tb.N, Tb.Th and Ct.Th and reduced Tb.Sp significantly in CKO group. Meanwhile, the H&E staining images of major visceral organs (heart, liver, spleen, lungs, and kidneys) from different mice groups also demonstrated that colivelin showed no significant biological toxicity (Fig. S6). All in all, these data illustrated that pharmacological activation of STAT3 by colivelin effectively reversed the decreased osteogenic ability of BMSCs and enhanced the bone volume of femurs from *Osx^Cre^; Igf2^fl/-^* mice.

## Discussion

Silver-Russell Syndrome (SRS) is a clinically heterogeneous disorder, which independently reported by Silver and Russell in the 1950s. While Silver described its symptoms as short stature, Russell noticed that the cranio-facial deformities were also associated with SRS 19,20, suggesting its close relationship with bone development. Recently, with the development of molecular genetic techniques, a general consensus has been reached in this field apart from its typical clinical manifestations 21. The loss of methylation of chromosome 11p15 serves as the most common etiologies of SRS accounting for approximately 60% of the cases, followed by pathogenic variants in imprinted genes, like IGF2 22. Moreover, the dysfunction of methylation also results in the reduced paternal IGF2 23, which highlights the importance of IGF2 in the SRS-related deformities, albeit the underlying mechanism still remains largely unexplored. Given that the skeletal deformities of SRS syndrome are particularly pronounced, it is necessary to investigate the expression pattern of IGF2 in the bone marrow niche and whether it also contributes to the syndrome.

As a special member of IGF family,* Igf2* is an imprinted gene, the expression of which depends on the paternal alleles via epigenetic regulations 24,25. It has been well established that *Igf2* has lots of physiology functions in anti-inflammation, metabolism, pro-proliferation and *etc* 26-28. In contrast to *Igf1* which maintains relatively high in circulation, *Igf2* expression decreases rapidly in circulation and many tissues after birth in human and mice 29. Nevertheless, the declining rate of *Igf2* varies among different tissues and IGF2 still exerts remarkable impacts on the maturation, homeostasis and function of central nervous, digestive and skeletal systems via autocrine and paracrine 30-33. Despite numerous studies indicating that locally derived IGF2 plays a pivotal role in the skeletal system, the details about how it works and what kinds of cells matter in this process still requires further study, impeding the comprehensive understanding of SRS.

In this study, to explore the question, the scRNA-seq was performed to explore the expression pattern of *Igf2* in different BM-resident cell. The results indicated that *Igf2* was predominantly expressed in mesenchymal cells and more enriched in SSPCs, similar to the expression pattern of *Igf1* 34,35. Various studies have noticed the distinct role of mesenchymal lineage cells in secreting cytokines, growth factors or vesicles, which is consistent with our analysis 36,37. Moreover, complex interactions were observed between SSPCs and pre-OBs in Igf2-lgf1r and Igf2-lgf2r signaling pathway, indicating the less differentiated mesenchymal cells may influence the differentiation and function of pre-OBs via IGF2. Next, to elucidate the function of *Igf2* in different OB lineage cells, we established three conditional-knockout mice model based on the pseudo-temporal order. As aforementioned,* Igf2* is expressed exclusively by the paternal allele, making it possible to eliminate* Igf2* expression in offspring as long as the mutated paternal allele is inherited 38. Thus, *Osx^Cre^*,* Prx1^Cre^* and *Ocn^Cre^* mice were selected to identify different clusters of SSPCs and OBs according to previous studies 39-41. We deleted *Igf2* from pro-OBs, BMSCs and OBs by crossing male *Igf2 flox* with female *Osx^Cre^*,* Prx1^Cre^* and *Ocn^Cre^* mice respectively and found significant bone deformities in the first two CKO mice. The femur of *Osx^Cre^; Igf2^fl/-^* exhibited varying degrees of bone malformations at different developmental stages. Similar phenomena were also observed in* Prx1^Cre^; Igf2^fl/-^* mice, but not *Ocn^Cre^; Igf2^fl/-^* mice at 12 weeks. These data highlighted SSPC-derived IGF-2 promote the development and homeostasis of skeleton as an autocrine factor, with its influence extending from the juvenile stage to adulthood, demonstrating although the overall expression of *Igf2* plummets with age, IGF2 can still exert its effects via autocrine and paracrine in skeletal system. Plus, this function of IGF2 can also partially account for the SRS-related skeletal deformities.

SSPCs actually represent a heterogeneous population, with their precise molecular identity remaining incompletely defined 42,43. Despite this ambiguity, SSPCs are increasingly recognized as pivotal regulators of skeletal homeostasis, serving as a dynamic reservoir for osteoblasts and chondrocytes. Our findings underscore the indispensable role of SSPC-derived IGF2 in maintaining this balance through STAT3 signaling 44,45. The depletion of *Igf2* in SSPCs disrupts osteoblast differentiation, leading to trabecular thinning and cortical defects, which somehow mirrors the age-related osteoporosis caused by damaged SSPCs 45. Importantly, the identification of IGF2-STAT3 as a critical axis in SSPC-osteoblast crosstalk reinforces the functional significance of these cells in bone remodeling. This mechanistic insight not only highlights SSPCs as central players in skeletal development but also positions them as a therapeutic target for disorders like SRS, where impaired osteogenesis drives pathology. Collectively, our work provides compelling evidence for SSPCs' indispensable role in bone biology.

It is well-acknowledged that the skeletal system undergoes lifelong remodeling process including the bone formation by osteoblasts and bone resorption by osteoclasts. In our analysis, the bone deformities of *Osx^Cre^; Igf2^fl/-^* and* Prx1^Cre^; Igf2^fl/-^* mice were caused by impaired bone formation while bone resorption remained intact.

Interestingly, previous studies indicated that IGF2 regulated the fate of mesenchymal progenitors, as well as the formation of giant osteoclasts, while others reported that IGF2 promoted osteogenic differentiation only in the presence of BMP-9 46,47. These results somehow contradict ours, potentially due to the limitation in the effective distance of autocrine and paracrine IGF2 47. Meanwhile, we have observed skull defects and condyle deformities as well, further demonstrating the locally derived IGF2 exerts impacts on skeletal system throughout the body (Fig. S3A, B). As the growth center of mandible, does the malformation of condyle account for SRS-related craniofacial deformities such as micrognathia 48,49? In depth investigation is needed.

Next, to discover the reasons for the substantial decline in osteogenic ability observed in *Osx^Cre^; Igf2^fl/-^*, we needed to select a representative mesenchymal cell cluster for further analysis. CD200 and PDGFR both have long been recognized as markers for mesenchymal progenitor cell and then the scRNA-seq data indicated that the expression landscape of the former is more consistent with that of *Igf2* at cellular level (Fig. S5A, B) 50,51. Thus, CD200 was chosen as the marker for the selection of mesenchymal cells.

Then following RNA-seq analysis indicated that JAK-STAT signaling pathway and *stat3* were significantly downregulated in the CD200^+^ cells from *Osx^Cre^; Igf2^fl/-^* mice compared with that from littermates. STAT3, a member of the Janus kinase-signal transducers and activators of transcription (JAK-STAT) family, responds to different kinds of growth factors and cytokines, thereby exerting a pivotal function in cell proliferation, differentiation, migration and survival 52-54. Its close relationship with skeletal system has been well-documented by various studies. Zhou *et al.* have systematically analyzed the skeletal deformities of mice deleted *Stat3* in osteogenic lineage cells and osteoclasts and found significant reduction in bone volume and quality in *Osx^Cre^; Stat3^fl/fl^* mice and *Prx1^Cre^; Stat3^fl/fl^* mice were dwarfed relative to their control littermates 18, while little difference was observed in *Ctsk^Cre^; Stat3^fl/fl^* mice. Meanwhile, *in vitro* studies also demonstrated its unique function in promoting the osteogenesis of osteoblasts 55-57. Moreover, previous studies indicated that the downregulation of *Igf2* also led to the inactivation of STAT3 58. Our results also verified its agonist, colivelin promoted osteogenic ability of BMSCs and rescued the bone loss phenotypes in *Osx^Cre^; Igf2^fl/-^* mice. These data illustrate the pivotal role of STAT3 as a downstream effector of IGF2 in the process of osteogenesis, consistent with previous research results. Meanwhile, our study highlights a promising future for the treatment of the SRS-related skeletal deformities through the regulation of STAT3, though more clinical studies are needed.

While this study provides mechanistic insights into the role of SSPC-derived IGF2 in skeletal development via STAT3 signaling, several limitations warrant consideration. First, interspecies differences between murine and human bone biology, such as variations in growth plate dynamics, hormonal regulation, and skeletal maturation timelines, may limit the direct translation of findings to human SRS 59. Second, scRNA-seq data, while informative, are subject to technical biases such as transcript dropout effects, particularly for low-abundance genes, potentially underrepresenting its spatial expression patterns 60. Additionally, the STAT3 agonist colivelin, though effective in rescuing osteogenic defects, may engage non-canonical pathways not fully characterized here, necessitating future studies to clarify its specificity. Besides, our focus on skeletal remodeling from 4wk-old to 8wk-old mice leaves unanswered questions regarding the long-term efficacy and safety of STAT3 activation in adult bone homeostasis or repair. Lastly, prior studies demonstrated that IGF2 activates PI3K/AKT and MAPK pathways as downstream of IGF2 in osteoarthritis and cancer to promote proliferation, survival, and disease progression 61-63. More investigations are needed for their potential tissue-specific signaling crosstalk or stage-dependent pathway activation. All in all, to address these limitations, future work should integrate human-derived cell lines, clinical SRS patient biopsies and long-term evaluation of colivelin administration to validate murine findings in human contexts, while employing multi-omics approaches to refine mechanistic understanding and optimize therapeutic strategies.

In conclusion, our research has clarified that SSPC-derived IGF2 supports the development and homeostasis of skeletal system, as well as revealed its mechanism. The deletion of *Igf2* in SSPCs (Prx1^+^ and Osx^+^), not osteoblasts (Ocn^+^) lead to the SRS-related bone deformities via downregulating *Stat3* and can be rescued by the activation of STAT3, which offers a therapeutic approach for treating skeletal disorders in SRS patients by modulating the JAK-STAT pathway.

## Methods

### ScRNA-sequencing

To investigate the expression pattern of *Igf2*, we selected scRNA-seq data published previously (accession code: GSE122467) 16. The cells from total mouse bone marrow (BM) were identified in the study and we divided cell groups at a more general level, namely mesenchymal cells, hematopoietic cells, endothelial cells and neuronal cells, to roughly locate in which major type of cells the* Igf2* was most expressed. Then to clarify the expression of *Igf2* in different mesenchymal cells, another scRNA-seq data was analyzed from GEO database (GSE138689), which focused on the mesenchymal cells (PRX1^+^ or LEPR^+^ cells). The scRNA-seq data was analyzed by Personal Biotechnology Co., Ltd. (China) and followed the companies' instructions.

### Mice

The *Igf2 flox* mice used in experiment were purchased from Jackson Laboratory and *Osx^Cre^*, *Prx1^Cre^* and *Ocn^Cre^* were purchased from Shanghai Model Organisms Center. All mice used in this study were kept with a C57BL/6 genetic background. They were caged individually in SPF standardization room with specific temperature (23 ± 2°C) and humidity (55 ± 5°C) on a 12:12 h light/dark cycle. All procedures were approved by the Animal Ethics Committee of the Affiliated Stomatology Hospital of Tongji University ([2022]-DW-11).

### Mice treated with STAT3 agonist colivelin

For agonist administration, 4-wk-old male *Osx^Cre^; Igf2^fl/-^* were randomly divided into two groups. Colivelin (MCE, HY-P1061A) at a concentration of 1mg/kg or equal volume of PBS was intraperitoneally injected respectively each other day for 4 weeks. Then, all mice were euthanized, and their femurs were subjected to both radiological and histological analyses.

### Primary BMSC isolation and culture

Adult male C57BL/6 mice were euthanized with an overdose of anesthetic and sterilized by immersion in 75% ethanol for 10 minutes. Under sterile conditions, femurs and tibias were dissected, and the epiphyseal ends were removed. The bone marrow was flushed from the cavities with 5 mL of α-minimum essential medium (α-MEM with 10% FBS, 100 mg/mL streptomycin, and 100 U/mL penicillin) using a syringe and were dissociated with enzymatic digestion solution: 3 mg/mL type I collagenase (Worthington, LS004197), 1 mg/mL Dispase II (Sigma, 04942078001) and 100 μg/mL DNase I (Sigma, D4527) in HBSS. After enzymatic digestion in water bath (37℃) for 15min, the process was stopped by resuspending in staining buffer (HBSS with 2% FBS). The collected marrow was filtered through a 200-mesh nylon filter and centrifuged at 300 g for 10 minutes at room temperature. The resulting pellet was resuspended, and the cells were seeded into culture dishes with medium containing α-MEM (Gibco, C12571500BT), 20% FBS (Gibco 10270-106, Lot: 42F6480K) and 1% penicillin/streptomycin (Gibco, 15-140-122). Cells were passaged when they reached confluence sufficient to form a distinct number of colonies and medium was changed on the next day and every 3 days thereafter.

### BMSC osteogenic differentiation

Primary BMSCs were expanded through two passages, then plated in a 24-well plate at 2×10^5 cells/well and cultured in complete α-MEM medium supplemented with 10% FBS and 1% penicillin-streptomycin. When 80% confluence were reached, the complete medium was replaced by osteogenic medium containing 1% (v/v) L-glutamine, 50 μg/mL (w/v) ascorbic acid, 10 mM β-glycerophosphate and 100 nM dexamethasone (all from Sigma-Aldrich). In addition, whether STAT3 agonist colivelin and vehicle (PBS) was added to the osteogenic medium or not was based on the experiment design. The BMSCs were induced for different days based on the following experiments.

### Alkaline phosphatase staining (ALP) and alizarin red staining (ARS)

The BMSCs were fixed in 4 % PFA for 30 mins after 7 days cultured in osteogenic medium with or without the agonist, colivelin. Afterwards, cells were rinsed with distilled water and subsequently stained utilizing the ALP Assay Kit (Beyotime Institute of Biotechnology, P0321S) in accordance with the manufacturer's instructions. Following incubation at 37°C for 30 min, the reaction was quenched using distilled water. Likewise, cells were undergone fixation and rinse after 21 days cultured in osteogenic medium. Alizarin Red Solution (OriCell, ALIR-10001) was utilized to detect the mineralized nodes. Distilled water was used to quench the reaction following a 30-min incubation at 37°C.

### Micro-CT analysis

Femur, skull, and mandible were scanned at 10 μm/slice resolution using a Micro-CT 50 scanner (Scanco Medical, Switzerland) set to 70 kVp and 124 μA. Three-dimensional reconstruction and analysis were conducted using Scanco evaluation software. For the trabecular bone parameters, the distal metaphysis of femurs was measured. The region of interest (ROI) was defined as the area below the distal growth plate where the epiphyseal cap structure was completely resorbed, extending for 100 slices towards the proximal femur. To accurately identify the trabecular bone in the metaphyseal region, we manually delineated its contour a few voxels away from the endocortical surface. For the cortical bone parameters, 100 slices from the mid-diaphysis of the femur were evaluated. Measurements including bone volume fraction (BV/TV), trabecular thickness (Tb.Th), trabecular separation (Tb.Sp), trabecular number (Tb.N), connectivity density (Conn.D), bone mineral density (BMD), structural model index (SMI), etc were calculated for quantification.

### RNA extraction and RT-qPCR

Total RNA was extracted from* in vitro* cells or bone tissue using the Trizol method (Takara, Japan). Briefly, chloroform was added to the Trizol solution, and the mixture was centrifuged at 12,000 rpm for 20 minutes at 4°C. The resulting supernatant was combined with an equal volume of isopropanol and centrifuged at 12,000 rpm for 15 minutes at 4°C. The RNA pellet was washed twice with 75% ethanol and then centrifuged at 8,000 rpm for 5 minutes at 4°C. RNA was reverse-transcribed to cDNA using the PrimeScript RT reagent kit with gDNA Eraser (Takara, Japan) in 20 μL reactions according to the manufacturer's protocol. RT-qPCR was performed utilizing the FastStart Essential DNA Green Master Kit (Roche, Basel, Switzerland) on a LightCycler 96 System (Roche). The primers used in this study were shown in Table S1.

### Protein extraction and Western blotting

Proteins were extracted from BMSCs using radio-immunoprecipitation assay (RIPA) lysis buffer (Sigma-Aldrich, St. Louis, MO, USA), supplemented with phosphatase and protease inhibitors. The protein samples were then reduced and denatured, followed by separation using 10% sodium dodecyl sulfate-polyacrylamide gel electrophoresis (SDS-PAGE). After protein separation by electrophoresis, PVDF membranes were used for transfer. The membranes were then blocked with 5% non-fat milk/TBST and probed with primary antibodies overnight at 4°C. Primary antibodies include GAPDH (Affinity, AF7021), STAT3 (CST, 9139), and p-STAT3 (CST, 9145). Following primary antibody incubation, horseradish peroxidase (HRP)-conjugated secondary antibodies (Beyotime Institute of Biotechnology, A0208) were applied. Chemiluminescent signals were detected using the GE ImageQuant LAS system. Quantitative analysis was performed using ImageJ software (National Institutes of Health, USA).

### Histological analysis

After euthanizing the mice, perfusion was performed using pre-cooled PBS and 4% paraformaldehyde (PFA, Kingmorn). The femurs were then dissected, fixed in 4% PFA for 24 hours and decalcified in 10% EDTA (SCR) for 4 weeks. Subsequently, the samples were embedded in paraffin and sectioned into 5-µm slices. Hematoxylin and eosin (H&E, Beyotime Institute of Biotechnology, C0105S) staining was performed in accordance with the recommended protocols to examine the histological morphology. Masson's Trichrome Stain Kit (Solarbio, G1340) and the alkaline phosphatase (ALP) staining kit (Beyotime Institute of Biotechnology, P0321S) were employed to assess bone formation and osteoblast activity. Tartrate-resistant acid phosphatase (TRAP) activity was tested by Sigma-Aldrich Acid Phosphatase kit (Sigma-Aldrich, 387A-1KT) to evaluate the activities of osteoclasts in the bone resorption area.

### Enzyme-linked immunosorbent assay (ELISA)

Blood samples from different groups were left at room temperature for 0.5 hour, followed by centrifugation at 3500 rpm for 10 minutes. The supernatant serum was retained and the precipitate was discarded. The serum was stored at -80°C until all samples were prepared for analysis. To assess bone formation and resorption activity, we measured the serum protein levels of pro-peptide of type I procollagen (PINP), C-terminal telopeptides of type I collagen (CTX-I), receptor activator NF-kappa B ligand (RANKL), and osteoprotegerin (OPG) using ELISA kits (mlbio, China), in strict accordance with the manufacturer's instructions.

### Immunofluorescence staining

For immunofluorescence staining, tissue sections were first deparaffinized and hydrated as described in histological analysis. Then, antigen retrieval was performed by treating the sections with hyaluronidase (H3506, Sigma-Aldrich) for 1 hour at 37°C. To block nonspecific background staining, sections were incubated with commercial goat serum (MXB, SP KIT-B2) for 1 hour at 37°C. Next, the sections were incubated with primary antibodies overnight at 4°C. The following primary antibodies were used: rabbit anti-osteopontin (OPN) (1:200, AF0227, Affinity), rabbit anti-osteocalcin (OCN) (1:200, Affinity, DF12303). The secondary antibody (anti-rabbit IgG A-11072, 1:1000, Invitrogen) then was incubated after 3 times of rinse by PBS at room temperature for 45 mins, followed by nuclear counterstaining with 4′,6-diamidino-2-phenylindole (DAPI, 2 µg/mL, Sigma-Aldrich) for 10 minutes. Finally, the sections were mounted using an anti-fade prolong reagent (Invitrogen), and images were captured.

### Calcein labeling

Mice were administered two intraperitoneal injections of calcein (Sigma, C0875-5G, 1 mg/ml in 2% NaHCO_3_ solution) at a dose of 20 mg/kg on days 0 and 7, and sacrificed on day 9. Following euthanasia, femurs were fixed in 4% paraformaldehyde (PFA) for 48 hours at 4°C, then rinsed in PBS. The bones were subjected to gradient dehydration in 15% and 30% sucrose solutions respectively for two nights at 4°C. Subsequently, femurs were embedded in OCT compound for cryosectioning and 20-μm-thick hard section was performed with the tissue sectioning and grinding system (EXAKT) for further analyses.

### Flow cytometry sorting and RNA-sequencing

For the analysis of mesenchymal lineage cells, the *Osx^Cre^; Igf1^f/-^* and littermate mice (8w, n=4 per group) were sacrificed and their femurs were dissected. The femurs were crushed with sterile scissors and digested in HBSS containing 3 mg/mL type I collagenase (Worthington, LS004197), 1 mg/mL Dispase II (Sigma, 04942078001) and 100 μg/mL DNase I (Sigma, D4527) at 37 °C for 15 min in a shaking bath for 2 rounds. Then, the digestion was terminated by adding double volume of stain buffer (HBSS containing 2% FBS) and the cells were filtered with a 200-mesh nylon filter. After centrifugation at 4 °C at 300 × g for 10 min, the supernatant was discarded and cells were suspended in red blood cell lysis buffer (00433357, eBioscience) at 4 °C for 5 min. Then, after neutralization and centrifugation, the cells were resuspended by staining buffer containing the CD16/32 blocking antibody (101320, Biolegend) and incubated for 30 min, followed by staining with antibodies. Antibodies used in our study including: anti-mouse CD45-APC (30-F11, Biolegend), anti-mouse TER119-APC (116212, Biolegend), anti-mouse CD31-APC (102409, Biolegend), anti-mouse CD200-PE/Cyanine 7 (123817, Biolegend). Every group of cells extracted from one mouse were added with 1 mL Trizol (Takara, Japan) and sent to Sinotech Genomics (China) for the subsequent sequencing.

### Statistical analysis

All quantitative data were presented as mean ± SD for absolute values, based on at least three replicates per group. All quantitive analyses of histomorphometry above were utilized the ImageJ software (National Institutes of Health, Bethesda, MD). Data normality was first assessed using the Shapiro-Wilk test and homogeneity of variance was verified via Levene's test. For comparisons between two groups, Student's t-test was applied, while one-way ANOVA with Tukey's post hoc correction was used for multi-group analyses by the GraphPad Prism software 5.0 (GraphPad Software, San Diego, CA). A statistical probability of p < 0.05 was considered significant.

## Supplementary Material

Supplementary figures and table.

## Abbreviations

IGF2: insulin-like growth factor 2
SRS: Silver-Russell Syndrome
SSPC: skeletal stem/progenitor cell
OB: osteoblast
OC: osteoclast
STAT3: signal transducers and activators of transcription 3
BMD: bone mineral density
BV/TV: bone volume ratio
Tb.Th: trabecular thickness
Tb.N: trabecular number
Conn.D.: connectivity density
Tb.Sp: trabecular spacing
SMI: structure model index
N.Ob/BS: Osteoblast number /bone surface
N.Oc/BS: osteoclast number /bone surface
PINP: pro-peptide of type I procollagen
CTX-I: C-terminal telopeptides of type I collagen
RANKL: receptor activator NF-kappa B ligand
OPG: osteoprotegerin
