# Supplementary material for: Skeletal stem/progenitor cell-derived rather than osteoblast-derived IGF2 supports the development and homeostasis of skeletal system via STAT3
Source: Int J Biol Sci. 2025 Apr 28;21(7):3229–46. doi: 10.7150/ijbs.112605 (PMC12080382; doi:10.7150/ijbs.112605)
Supplement: Supplementary file 1 — Supplementary figures and table. [file ijbsv21p3229s1.pdf]

Figure S1 The expression pattern of the *Igf2bps* (*Insulin-like growth factor 2 binding proteins*), *Igfbps* (*Insulin-like growth factor binding proteins*) and *Igf2r* in mesenchymal cells.

A~I t-SNE plot showing the expression pattern of *Igf2bp1, Igf2bp2, Igf2bp3, Igfbp2, Igfbp3, Igfbp4, Igfbp5, Igfbp6* and *Igf2r* among mesenchymal cells.

Figure S2 Deletion of *Igf2* in pro-osteoblasts induces significant lower expression of *Igf2* and osteogenesis-related genes in CKO mice.

A RT-qPCR showing the relative expression of *Igf2* of BMSCs from Osx^{Cre} (Ctrl) and Osx^{Cre}; $Igf2^{fl/-}$ (CKO) mice (n=3 per group).

B RT-qPCR showing the relative expression of *Col1a1*, *Alp*, *Runx2*, *Sp7* and *Ocn* of BMSCs from Osx^{Cre} (Ctrl) and Osx^{Cre}; $Igf2^{fl/-}$ (CKO) mice (n=3 per group).

Figure S3 Deletion of *Igf2* in pro-osteoblasts induces in structural deformities in craniofacial bones in CKO mice.

A Representative micro-CT images of mandibles and their magnifying views of the condyles from 12-wk-old male Osx^{Cre}; $Igf2^{fl/-}$ (CKO) mice and littermate controls (Ctrl). Scale bar=2mm for left views and 500μm for magnified views.

B Representative micro-CT images of skulls from 12-wk-old male Osx^{Cre}; $Igf2^{fl/-}$ (CKO) mice and littermate controls (Ctrl). The yellow arrows point to the structural deformities of the CKO mice. Scale bar=2mm.

Figure S4 The skeleton maldevelopment also exists in Osx^{Cre}; $Igf2^{fl/-}$ mice in earlier developmental stage.

A Representative micro-CT images of femur and its magnifying views of the femur sections from 4-wk-old male Osx^{Cre}; $Igf2^{fl/-}$ mice and littermate controls. Scale bar=1mm.

B Statical analysis of micro-CT of trabecular bone volume ratio (BV/TV), trabecular thickness (Tb. Th), trabecular number (Tb. N) and trabecular spacing (Tb. Sp) (n=4 per genotype).

C Representative micro-CT images of skulls from 4-wk-old male Osx^{Cre}; $Igf2^{fl/-}$ (CKO) mice and littermate controls (Ctrl). The yellow arrow points to the structural deformities of the CKO mice. Scale bar=2mm.

D Statical analysis of micro-CT of cortical bone volume ratio (BV/TV) in C (n=3 per genotype).

E Representative H&E and Masson trichrome staining images of the femur sections from 2-wk&4-wk-old male Osx^{Cre}; $Igf2^{fl/-}$ mice and littermate controls. Scale bar=500μm.

F Representative TRAP staining images of the femur sections from 2-wk&4-wk-old male Osx^{Cre}; $Igf2^{fl/-}$ mice and littermate controls (n=5 per genotype) and statistical analysis. Scale bar=100μm.

G Representative ALP staining images of the femur sections from 2-wk&4-wk-old male Osx^{Cre}; $Igf2^{fl/-}$ mice and littermate controls and statistical analysis (n=3 per genotype). Scale bar=100μm.

H Representative Immunofluorescence staining of OCN of the femur sections from 2-wk-old&4-wk-old male Osx^{Cre}; $Igf2^{fl/-}$ mice and littermate controls and statistical analysis (n=3 per genotype). Scale bar=100μm.

I Representative Immunofluorescence staining of OPN of the femur sections from 2-wk-old&4-wk-old male Osx^{Cre}; $Igf2^{fl/-}$ mice and littermate controls and statistical analysis (n=3 per genotype). Scale bar=100μm.

Figure S5 The expression pattern of mesenchymal markers and flow cytometry sorting before RNA-seq.

A, B The expression pattern of classic mesenchymal markers: *Cd200* and *Pdgfr*.

C Flow cytometry sorting showing the $Cd200^{+}$ and $CD45^{-}Ter119^{-}CD3^{-}$ cells were selected.

Figure S6 Biotoxicity analysis of colivelin via histopathological examinations

Representative H&E staining images of the heart, liver, spleen, lung and kidney from Osx^{Cre} (Ctrl) and Osx^{Cre}; $Igf2^{fl/-}$ (CKO) mice with vehicle or STAT3 agonist colivelin. Scale bar=100μm.

Supplementary Table 1. Primers' sequence used for RT-qPCR

Genes		Sequence
Igf2	Forward	5'-GTGCTGCATCGCTGCTTAC-3'
	Reverse	5'-ACGTCCCTCTCGGACTTGG-3'
Runx2	Forward	5'-ATGCTTCATTCGCCTCACAAA-3'
	Reverse	5'-GCACTCACTGACTCGGTTGG-3'
Alp	Forward	5'-CCAACTCTTTTGTGCCAGAGA-3'
	Reverse	5'-GGCTACATTGGTGTTGAGCTTTT-3'
Ocn	Forward	5'-AAATAGCCCTGGCAGATTCC-3'
	Reverse	5'-CAGCCTCCAGCACTGTTTAT-3'
Sp7	Forward	5'-ATGGCGTCCTCTCTGCTTG-3'
	Reverse	5'-TGAAAGGTCAGCGTATGGCTT-3'
Col1a1	Forward	5'-GCTCCTCTTAGGGGCCACT-3'
	Reverse	5'-CCACGTCTCACCATTGGGG-3'
Stat3	Forward	5'-CAATACCATTGACCTGCCGAT-3'
	Reverse	5'-GAGCGACTCAAACTGCCCT-3'
GAPDH	Forward	5'-AGGTCGGTGTGAACGGATTTG-3'
	Reverse	5'-TGTAGACCATGTAGTTGAGGTCA-3'

Figure S1

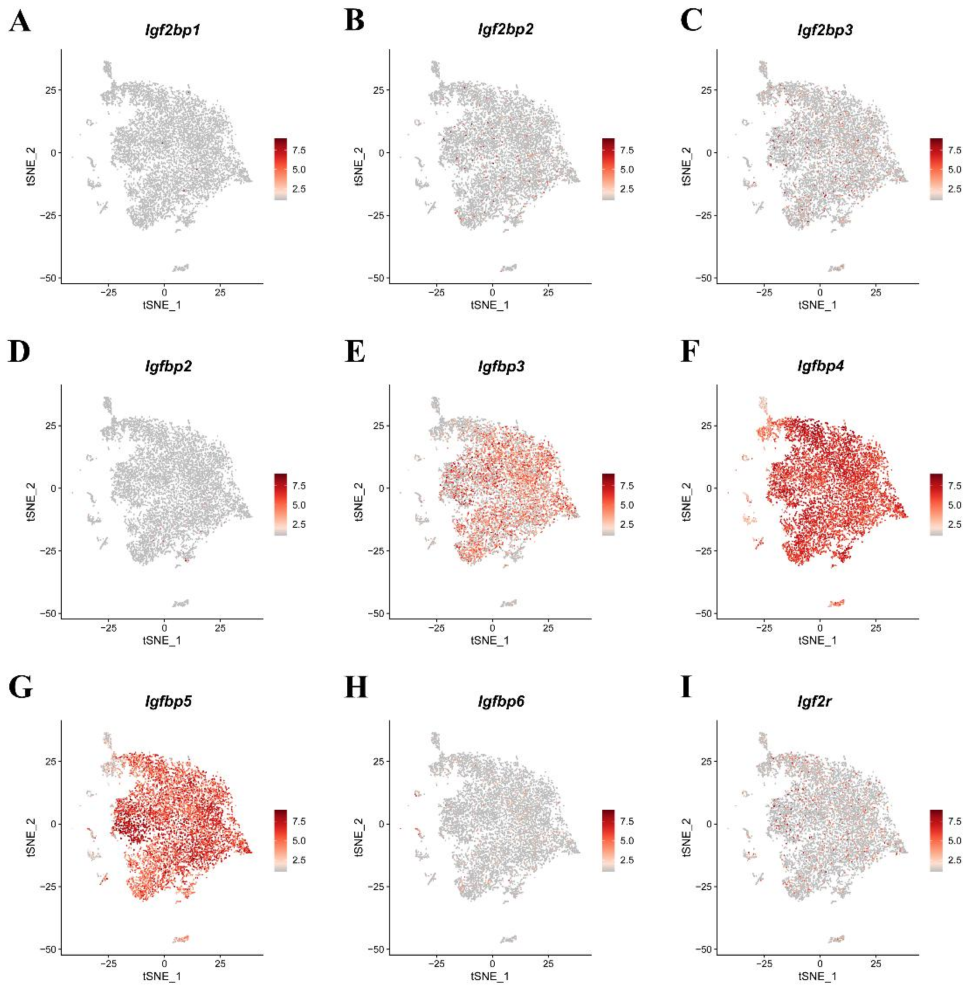
A
Igf2bp1
B
Igf2bp2
C
Igf2bp3
D
Igfbp2
E
Igfbp3
F
Igfbp4
G
Igfbp5
H
Igfbp6
I
Igf2r
tSNE_1
tSNE_2
7.5
5.0
2.5

Figure S2

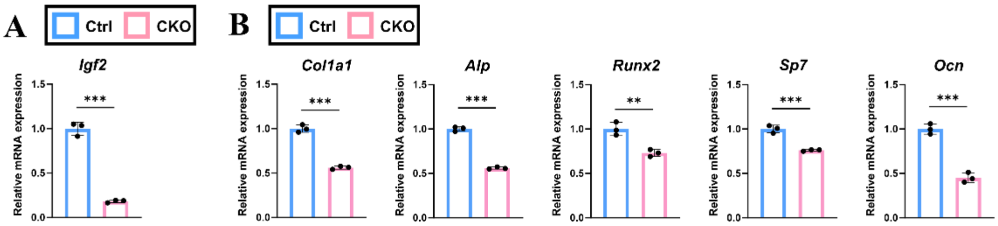
A
Ctrl
CKO
Igf2
Relative mRNA expression
1.5
1.0
0.5
0.0
***
B
Ctrl
CKO
Col1a1
Alp
Runx2
Sp7
Ocn
Relative mRNA expression
***
***
**
***
***

Figure S3

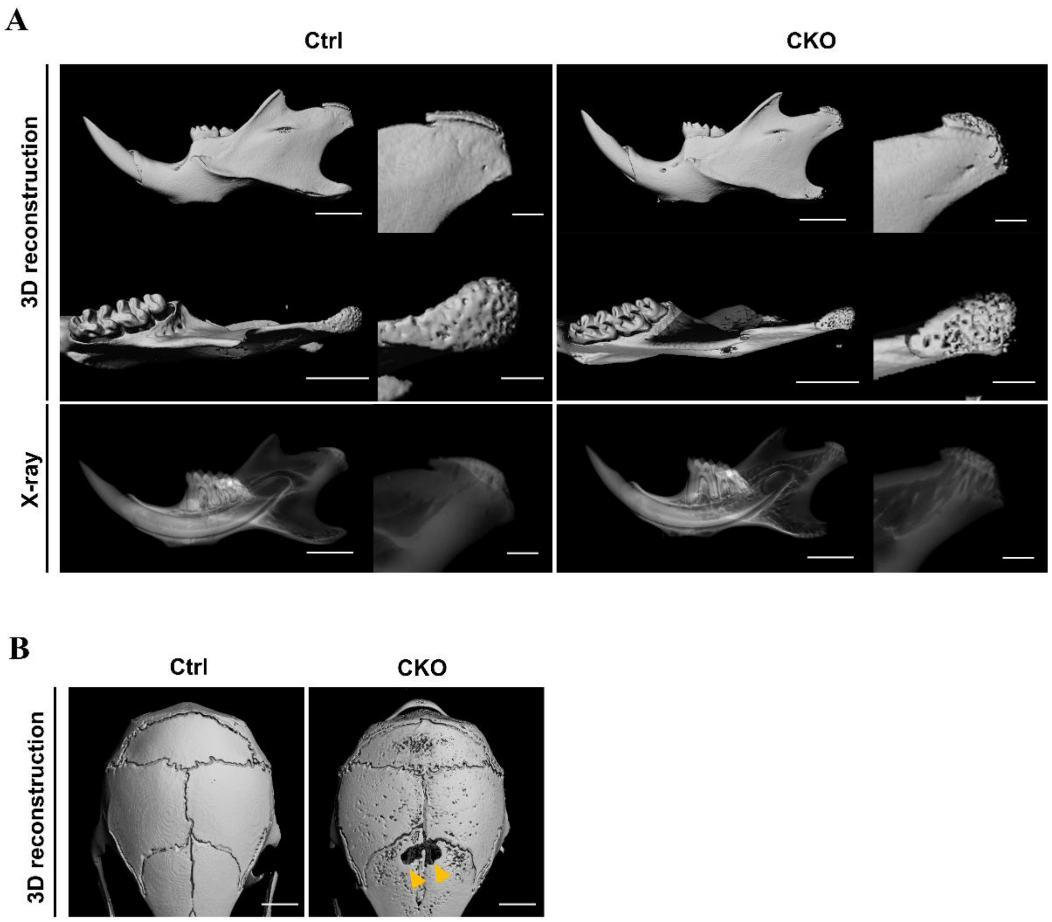
A
Ctrl
CKO
3D reconstruction
X-ray
B
Ctrl
CKO
3D reconstruction

Figure S4

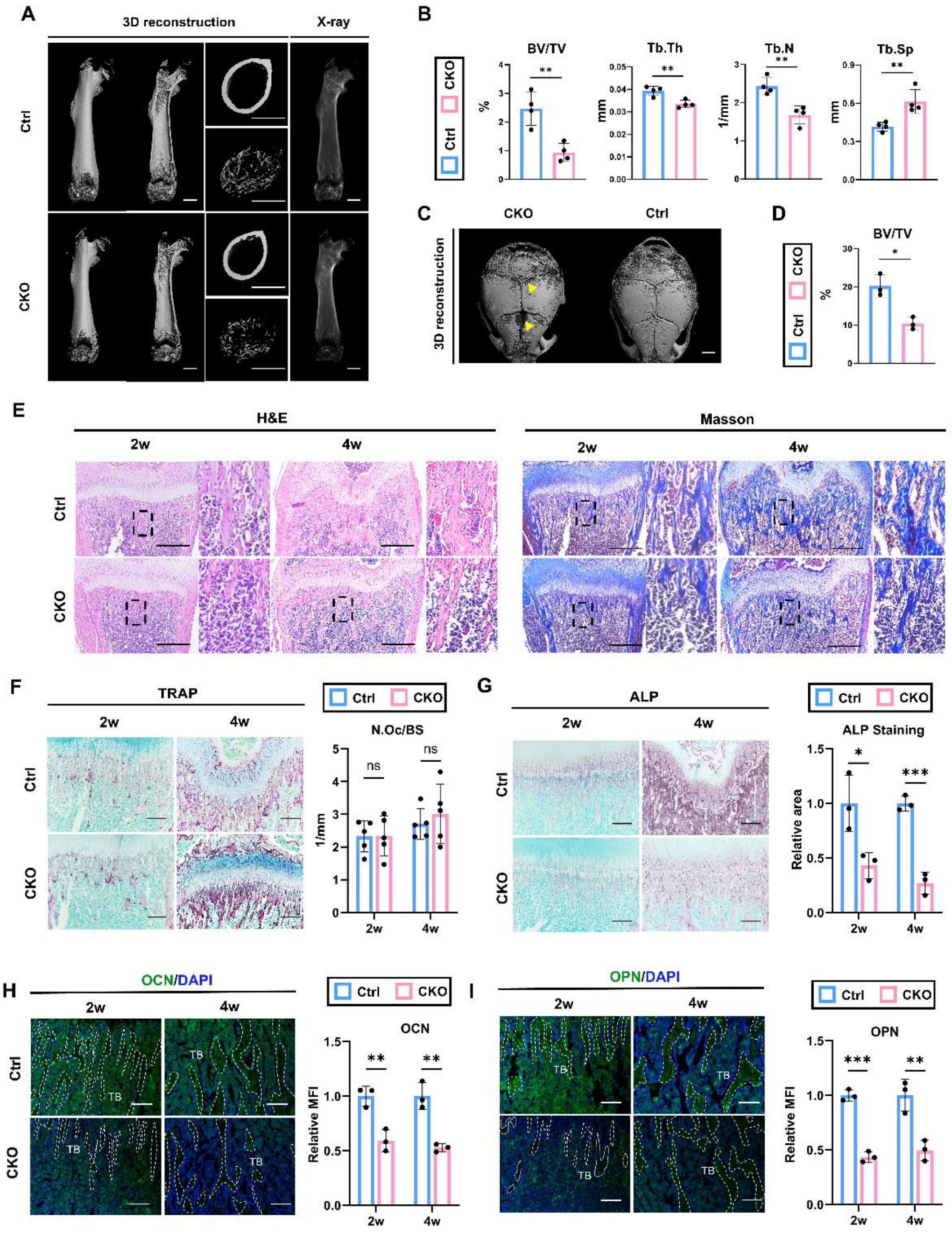
A
3D reconstruction
X-ray
Ctrl
CKO
B
BV/TV
Tb.Th
Tb.N
Tb.Sp
Ctrl
CKO
C
CKO
Ctrl
3D reconstruction
D
BV/TV
E
H&E
Masson
2w
4w
F
TRAP
N.Oc/BS
ns
G
ALP
ALP Staining
Relative area
H
OCN/DAPI
OCN
Relative MFI
TB
I
OPN/DAPI
OPN

Figure S5

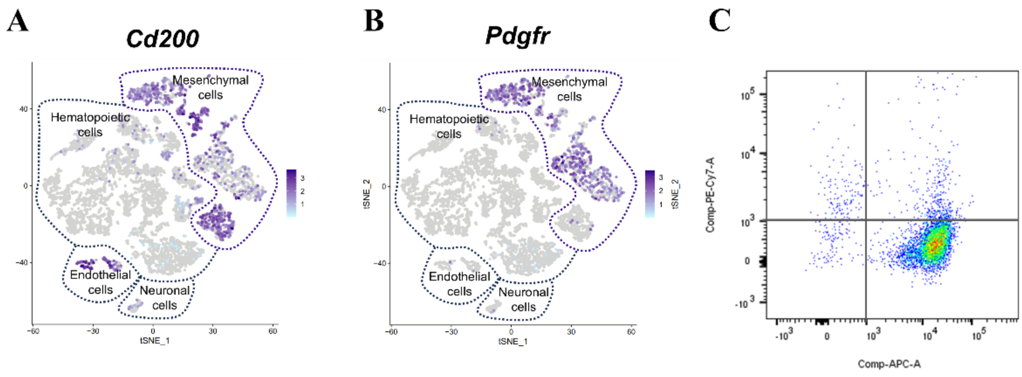
A
Cd200
Mesenchymal cells
Hematopoietic cells
Endothelial cells
Neuronal cells
tSNE_1
B
Pdgfr
tSNE_2
C
Comp-PE-Cy7-A
Comp-APC-A

Figure S6

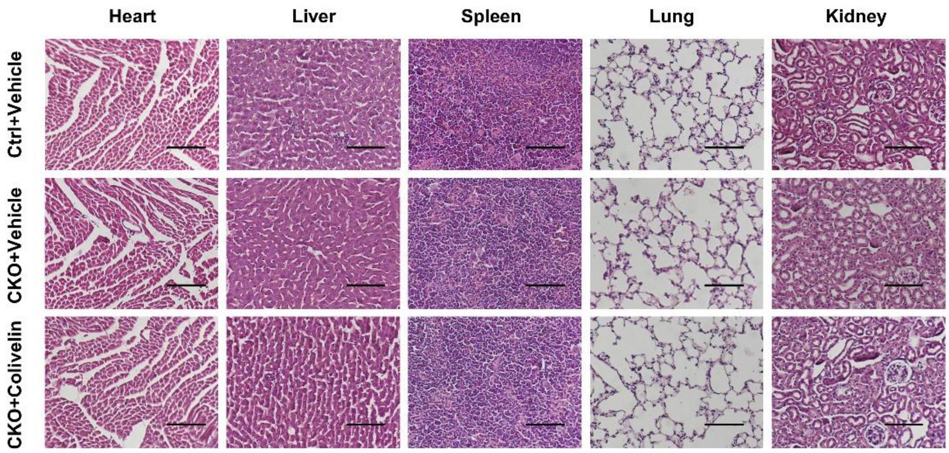
Heart
Liver
Spleen
Lung
Kidney
Ctrl+Vehicle
CKO+Vehicle
CKO+Colivelin
